# Recent Advances in Archaeal Translation Initiation

**DOI:** 10.3389/fmicb.2020.584152

**Published:** 2020-09-18

**Authors:** Emmanuelle Schmitt, Pierre-Damien Coureux, Ramy Kazan, Gabrielle Bourgeois, Christine Lazennec-Schurdevin, Yves Mechulam

**Affiliations:** Laboratoire de Biologie Structurale de la Cellule, BIOC, Ecole Polytechnique, CNRS-UMR7654, Institut Polytechnique de Paris, Palaiseau, France

**Keywords:** mRNA, Shine-Dalgarno, leaderless, ribosomal proteins, evolution

## Abstract

Translation initiation (TI) allows accurate selection of the initiation codon on a messenger RNA (mRNA) and defines the reading frame. In all domains of life, translation initiation generally occurs within a macromolecular complex made up of the small ribosomal subunit, the mRNA, a specialized methionylated initiator tRNA, and translation initiation factors (IFs). Once the start codon is selected at the P site of the ribosome and the large subunit is associated, the IFs are released and a ribosome competent for elongation is formed. However, even if the general principles are the same in the three domains of life, the molecular mechanisms are different in bacteria, eukaryotes, and archaea and may also vary depending on the mRNA. Because TI mechanisms have evolved lately, their studies bring important information about the evolutionary relationships between extant organisms. In this context, recent structural data on ribosomal complexes and genome-wide studies are particularly valuable. This review focuses on archaeal translation initiation highlighting its relationships with either the eukaryotic or the bacterial world. Eukaryotic features of the archaeal small ribosomal subunit are presented. Ribosome evolution and TI mechanisms diversity in archaeal branches are discussed. Next, the use of leaderless mRNAs and that of leadered mRNAs having Shine-Dalgarno sequences is analyzed. Finally, the current knowledge on TI mechanisms of SD-leadered and leaderless mRNAs is detailed.

## Introduction

**Figure 1:**
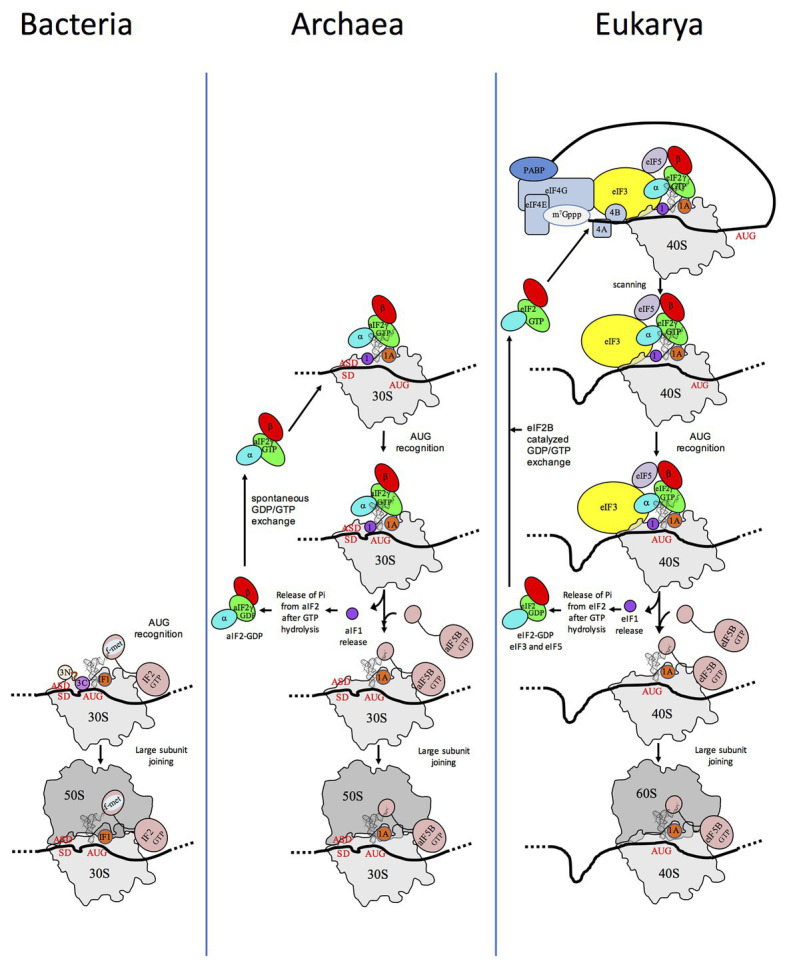
Schematic views of the translation initiation (TI) steps in the three domains of life. The figure illustrates the main steps in bacteria (left), in archaea (middle), and in eukarya (right). Bacterial 30S subunit recruits the messenger RNA (mRNA), often due to the base pairing between a Shine-Dalgarno sequence (SD) with an ASD sequence at the 3'-end of 16S rRNA. Three initiation factors, IF1, IF2, and IF3 favor the recruitment of the initiator tRNA and its pairing with the start codon. The formyl-methionyl moiety of the initiator tRNA is important for recognition by IF2. After start codon recognition, IF3 is released and the large ribosomal subunit is recruited with the help of IF2 (see Mechulam et al., 2011; Rodnina, 2018 for reviews). Archaea and eukarya share a common set of factors comprising e/aIF1A, e/aIF1, e/aIF2, and e/aIF5B (see also Figure 2). e/aIF2 heterotrimer is represented with a three-color code (α subunit in cyan, β subunit in red, and γ subunit in green). In canonical eukaryotic translation, a pre-initiation complex, containing the small ribosomal subunit, the methionylated initiator tRNA, and initiation factors, forms at the 5'-capped end of the mRNA. The complex then scans the mRNA until a start codon in a suitable environment is found. Base-pairing of the tRNA anticodon with the AUG start codon triggers eIF1 release followed by the release of Pi resulting from GTP hydrolysis by eIF2 (Algire et al., 2005). In turn, eIF2, eIF3, and eIF5 are released; eIF5B-GTP is recruited and favors joining with the large ribosomal subunit (see Hinnebusch, 2017 for a review). Archaea often use an SD sequence for mRNA recruitment. The 30S subunit is then definitely positioned with the start codon in the P site thanks to base-pairing with the tRNA anticodon. Overall, the four initiation factors aIF1, aIF1A, aIF2, and aIF5B play similar roles as their eukaryotic counterparts (see text and Schmitt et al., 2019 for a mechanism-oriented review). In the three cases, the translation competent IC is formed after the release of e/aIF1A (or IF1 in bacteria) and e/aIF5B (or IF2 in bacteria). In eukarya, the complex formed by eIF4E + eIF4G + eIF4A is known as eIF4F. eIF3, composed of 6 (yeast) to 13 (mammals) subunits is represented as a yellow oval. The figure is adapted from Schmitt et al. (2019).

Translation initiation (TI) allows accurate selection of the initiation codon on a messenger RNA (mRNA), which then defines the reading frame of the protein to be synthesized. In all domains of life, translation initiation generally occurs within a macromolecular complex made up of the small ribosomal subunit (SSU), the mRNA, a specialized methionylated initiator tRNA, and translation initiation factors (IFs). Once the start codon is selected at the P site of the ribosome and the large subunit is associated, the IFs are released and a ribosome competent for elongation is formed. However, even if the general principles are the same in the three domains of life, the molecular mechanisms are different in bacteria, eukaryotes, and archaea and may also vary depending on the mRNA (Figure 1).

In bacteria, mRNAs are not further processed after transcription and the 5' untranslated region (5'-UTR) often carries a “Shine-Dalgarno” (SD) sequence containing a GGAGG consensus complementary to the 3' end of the 16S rRNA of the SSU (Shine and Dalgarno, 1974). They can also be devoid of SD sequence or even have no 5′-UTR at all. The methionylated initiator tRNA is formylated and the formyl group is crucial for its accurate selection by the initiation complex (Guillon et al., 1993, 1996). Only three initiation factors are involved, IF1, IF2, and IF3 (for reviews see, for example, Mechulam et al., 2011; Rodnina, 2018).

**Figure 2:**
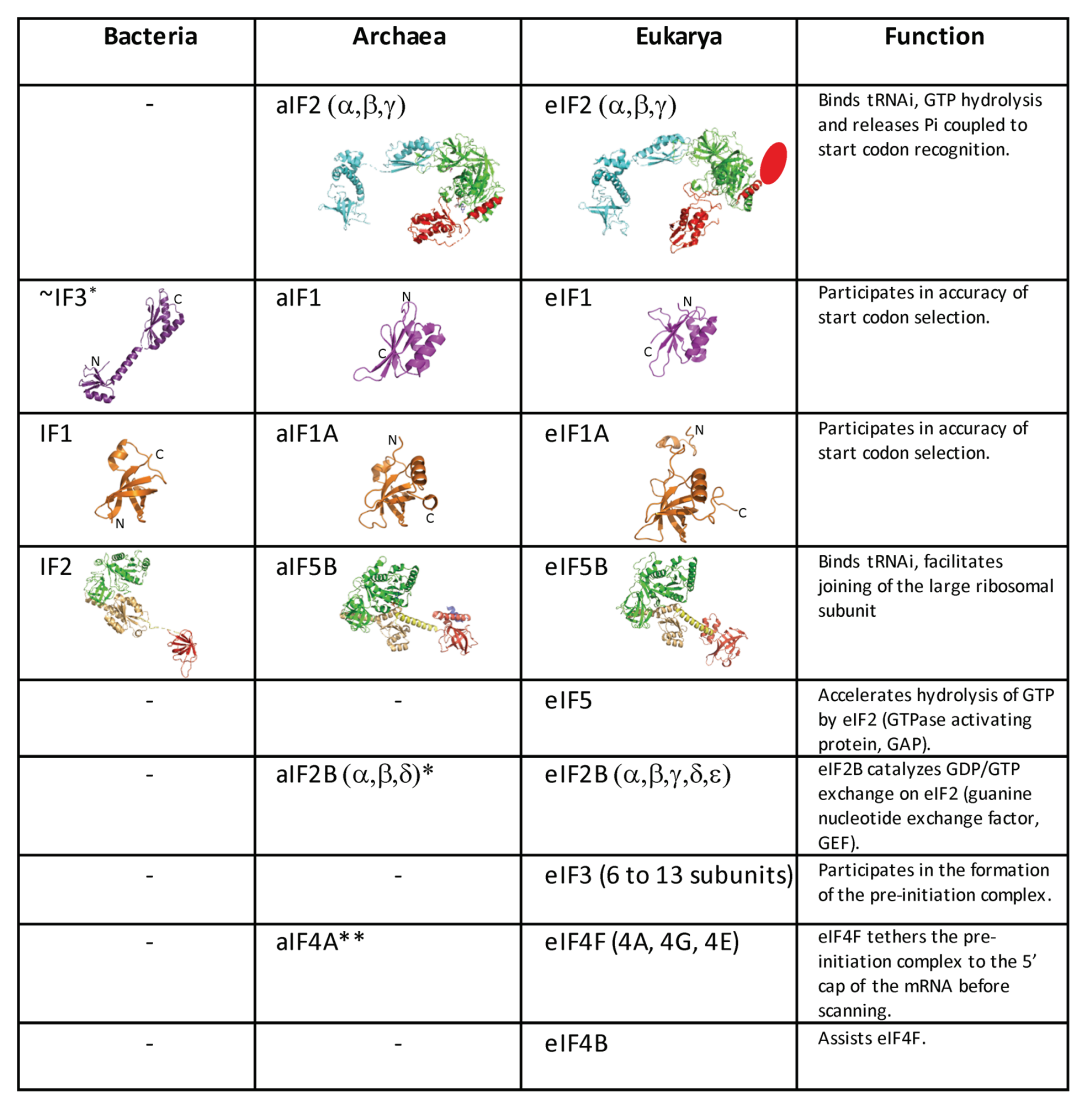
Translation initiation factors in the three domains of life. The structures of the archaeal translation initiation factors and of their orthologues in eukaryotes and bacteria (when present) are shown. e/aIF2 is colored as in Figure 1. The unknown structure of the N-domain specific of eukaryotic eIF2β is shown as an oval. The structure of aIF2 is from PDB 3V11 (Schmitt et al., 2012), those of aIF1 and aIF1A are from Coureux et al. (2016). The structures of eIF2, eIF1, and eIF1A are from PDB 6FYX (Llacer et al., 2018). IF1 is from PDB 3I4O (Hatzopoulos and Mueller-Dieckmann, 2010). Bacterial IF3 is a two-domain protein. The correspondence between IF3 and e/aIF1 is based on a structural and functional resemblance of the IF3 C-terminal domain with e/aIF1. Despite this resemblance, the topologies of the two α–β folds are different. This suggests that they do not derive from a common ancestor. aIF5B is from PDB 1G7T (Roll-Mecak et al., 2000), eIF5B is from PDB 4N3N (Kuhle and Ficner, 2014), and IF2 is from PDB 5LMV (Hussain et al., 2016). The color code for e/aIF5B/IF2 is as follows: G-domain and domain II in green, domain III in light orange, linker in yellow, and domain IV in red. The specific archaeal helix in domain IV is shown in blue. ^*^The catalytic γ and ε subunits of eIF2B are missing in archaea. The function of the eIF2B α, β, δ homologues in archaea is not clear and may be unrelated to translation initiation (Dev et al., 2009; Gogoi et al., 2016). ^**^The aIF4A orthologue is present in many archaea. However, deletion of the corresponding gene in *Haloferax volcanii* showed only a small phenotype (Gäbel et al., 2013).

In eukaryotes, translation initiation is more complicated with many IFs involved (Figure 2). mRNAs are maturated with a m^7^G-cap at the 5′ end and a polyadenylated tail at the 3′ end. The cap-dependent canonical translation initiation model involves a pre-initiation complex (43S PIC) containing the SSU, the ternary complex eIF2-GTP-Met-tRNA_i_^Met^, the two small factors, eIF1 and eIF1A, and two proteins with regulatory functions, eIF5 and eIF3. eIF5 is the activating protein for the eIF2 GTPase, and eIF3 is a large multimeric architectural protein involved in mRNA binding. In the presence of factors of the eIF4 family and of the poly(A)-binding protein (PABP) associated with the poly(A) tail of the mRNA, the 43S PIC is recruited at the 5'-cap extremity of the mRNA, thereby forming the 48S PIC. In mammals, direct interaction of eIF3 with eIF4F favoring the formation of the 48S complex was shown (Korneeva et al., 2000). However, these interactions were not detected in *Saccharomyces cerevisiae* (Jivotovskaya et al., 2006). The 48S PIC then scans the mRNA until an AUG codon in a correct context (Kozak motif) is found (Kozak, 1986). Recognition of the AUG codon stops scanning, causes the release of factors and the assembly of an 80S complex competent for elongation *via* the junction with the large subunit, using eIF5B and eIF1A (for a review see, for example, Hinnebusch, 2017). Besides this canonical mechanism, a certain number of alternative starting routes have also been described (Shatsky et al., 2018).

Archaeal TI harbors bacterial and eukaryotic features. In archaea, mRNAs are not further processed after transcription. They have Shine-Dalgarno sequences or very short 5′-UTR. Hence, archaeal mRNA features are close to bacterial mRNA features. In contrast, genomic analyses showed that archaeal initiation factors correspond to a subset of eukaryotic translation initiation factors. Indeed, aIF1, aIF1A, aIF2, and aIF5B homologous to the corresponding eukaryotic factors are present (Figures 1, 2; Dennis, 1997; Kyrpides and Woese, 1998; Benelli and Londei, 2011; Gäbel et al., 2013; Schmitt et al., 2019). Thus, even if there are obvious differences between archaea and eukaryotes, in particular for the recruitment of mRNA, *via* SD sequences vs. long-range scanning, the selection of the start codon is carried out within a same structural core composed of the small ribosomal subunit, mRNA, methionylated initiator tRNA (Met-tRNA_i_^Met^), and the three initiation factors e/aIF1, e/aIF1A, and e/aIF2 (Schmitt et al., 2019). Finally, the late steps of TI preceding the formation of a ribosome competent for elongation are controlled by initiation factors that are conserved in the three domains of life, IF1-e/aIF1A, and IF2-e/aIF5B.

Because TI mechanisms have evolved lately, their studies bring important information about the evolutionary relationships between extant organisms. In this context, recent structural data on ribosomal complexes and genome-wide studies are particularly valuable.

This review will focus on archaeal translation initiation highlighting its relationships with either the eukaryotic or the bacterial world. We first describe eukaryotic features of the archaeal small ribosomal subunit possibly related to TI mechanisms and discuss the diversity of the archaeal ribosome among archaeal phyla. Next, we discuss the occurrence of leaderless mRNAs and that of leadered mRNAs having Shine-Dalgarno sequences. The current knowledge on TI mechanisms of SD-leadered and leaderless mRNAs is then presented.

## The Archaeal Ribosome is of the Eukaryotic Type

**Figure 3:**
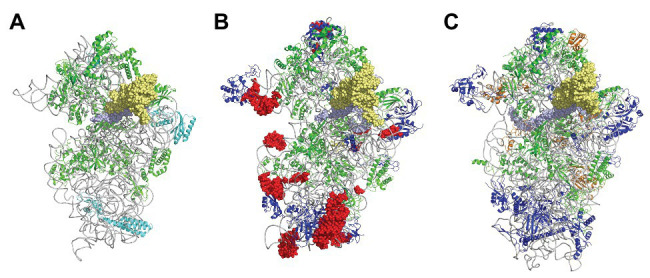
The small ribosomal subunit in the three domains of life. **(A)** 30S from *Thermus thermophilus* (PDB 5LMV; Hussain et al., 2016). **(B)** 30S from *Pyrococcus abyssi* (PDB 6SWC; Coureux et al., 2020). **(C)** 40S from *Kluyveromyces lactis* (PDB 6FYX; Llacer et al., 2018). Ribosomal proteins are colored as follows; universal in green, bacterial in cyan, eukaryotic and archaeal in dark blue, and eukaryotic only (as compared to *P. abyssi*) in orange. The P site tRNA is in yellow spheres and the mRNA in light blue spheres. rRNA is in gray. Regions of the ribosomal RNA of the *P. abyssi* small subunit that are not in the common core as defined in Bernier et al. (2018) are shown with red spheres (middle view, Table 2).

In the 1980s, Woese noted that rRNAs were excellent molecular chronometers that could be used to trace the molecular phylogenetic relationships between extant individuals. Indeed, rRNA are found in all organisms, are easily isolated and sequenced, and show positions that vary at different rates. Analysis of sequence/secondary structure variations in rRNAs allowed definition of Archaea as a third branch of the tree of life (Woese and Fox, 1977; Noller and Woese, 1981; Woese et al., 1983, 1990; Woese, 1987). Since these pioneering studies, many other works were dedicated to evolution of the ribosome (Cannone et al., 2002; Roberts et al., 2008; Fox, 2010; Petrov et al., 2015). Thanks to the increasing number of sequences and to the availability of high-resolution three-dimensional structures of ribosomes representative of each domain of life, evolutionary relationships between organisms appeared even clearer. A universal core making the structural and functional foundation of rRNAs of all cytoplasmic ribosomes was defined (Bernier et al., 2018). At the level of the SSU, this common core corresponds to 90% of bacterial rRNA and encompasses the decoding center of the small ribosomal subunit with in particular the 530 loop and the 1,490 region (*Escherichia coli* numbering) but not the 3' end corresponding to the mRNA exit region. Archaeal ribosomes have rRNA molecules very close in size to that of bacterial rRNAs explaining why the sedimentation coefficients of the archaeal ribosomal subunits are the same as that of bacterial ribosomes (Table 1). However, some regions are divergent (some of the divergent regions of the SSU colored in red in Figure 3), and rRNAs of archaeal ribosomes are closer to eukaryotic rRNAs than to bacterial rRNAs (Woese, 1987; Roberts et al., 2008; Bernier et al., 2018; Bowman et al., 2020; Penev et al., 2020).

**Table 1:** Ribosomes in the three domains of life.

Domain	Sedimentation coefficient		rRNA	Ribosomal proteins
Bacteria	70S	30S	16S (1493)	21 (15u, 6b)
		50S	23S (2891)5S (117)	33 (18u, 15b)
Archaea	70S	30S	16S (1483)	25 (15u, 9e, 1a)
		50S	23S (2967)5S(122)	39 (18u, 20e)
Eukarya	80S	40S	18S (1860)	33 (15u, 18e)
		60S	28S (4039)5S (120)5.8S (158; *S. cerevisiae*)	46 (18u, 28e)

Data concerning rRNA are from Bernier et al. (2018). Values in parentheses indicate the mean number of bases according to the SEREB (Sparse and Efficient Representation of Extant Biology) sampling except for the 5.8S rRNA. The number of species in the SEREB sample is 67 bacteria, 36 archaea, and 30 eukarya. Sequence alignments are accessible in Bernier et al. (2018). The ribosomal protein contents are indicated for *E. coli*, *S. cerevisiae*, and *P. abyssi*. These numbers slightly vary depending on the organism (Lecompte et al., 2002). The number of universal (u), eukaryotic (e), bacterial (b), archaeal (a) -type ribosomal protein is indicated. One protein possibly specific to archaea (a) has recently been identified in *P. abyssi* (Coureux et al., 2020; Nurenberg-Goloub et al., 2020).

Phylogenetic studies were further refined using ribosomal protein sequences. The archaeal ribosome contains ribosomal proteins (r-proteins) that are either universal (33 r-proteins) or specific to eukarya and archaea (29 r-proteins; Table 1). No r-proteins found only in the archaeal and bacterial domains are found. One protein that could be specific of the archaeal domain found in place of eukaryotic eS21 and, therefore, named aS21 was identified recently in the SSU of *Pyrococcus abyssi* (Coureux et al., 2020) and *Thermococcus celer* (Nurenberg-Goloub et al., 2020). However, further phylogenetic studies are required to firmly determine whether the protein is unique to archaea or distantly related to eS21. The 2014 system for naming ribosomal proteins is used throughout the manuscript. According to this naming, eukaryotic and archaeal specific proteins are named eSX or eLX (Ban et al., 2014). Unfortunately, this naming does not directly distinguish r-proteins that are either present in eukaryotes and archaea from those present only in eukaryotes. Given the growing importance of studies of the archaeal ribosome, a naming including an ae prefix for specifying archaeal and eukaryotic proteins would now be desirable.

Structurally invariable cores are found in universal proteins. However, in addition to the core, protein segments or extensions show late evolution reflecting specialization in the three domains of life (Melnikov et al., 2018). Concerning the 29 r-proteins specific to eukarya and archaea, it is interesting to note that some of these proteins contact regions of the 16S rRNA outside of the common core (red patches in Figure 3 and Table 2). Phylogenetic analysis of archaeal/eukaryotic specific r-proteins show that content in r-proteins vary depending on the archaeal branch (Lecompte et al., 2002; Hartman et al., 2006; Yutin et al., 2012). In particular, Crenarchaeota, Thaumarchaeota, and Korarchaeota have more r-proteins than Euryarchaeota and Nanoarchaeota. More recently, an Asgard superphylum close to the TACK one has been identified (Eme et al., 2017). Examination of the first Asgard genomes (Akil and Robinson, 2018; Imachi et al., 2020) also revealed a higher content of r-proteins as observed in TACK. For instance, TACK and Asgard SSU contain S25e, S26e, and S30e, whereas these proteins are not found in euryarchaea. Altogether, these findings agree with the proposed origin of eukaryotes from within an archaeal superphylum close to Asgard and TACK (Hartman et al., 2006; Guy and Ettema, 2011; Yutin et al., 2012; Williams et al., 2013; Eme et al., 2017; Zaremba-Niedzwiedzka et al., 2017; Castelle and Banfield, 2018; Melnikov et al., 2020). Notably, a recent study in Lokiarchaea and Heimdallarchaea further decreased the gap between eukaryotes and archaea by identifying eukaryotic-like expansion segments in large subunit rRNA in these archaea (Penev et al., 2020). As discussed below, the availability of high-resolution structures of functional states of ribosomes in the three domains of life now provide data for functional and structural comparisons leading to validation of sequence-based models.

**Table 2:** Regions of Pab-16S rRNA that are not in the common core and the r-proteins found nearby.

Regions of Pab-16S rRNA not in the common core	Archaeal and eukaryotic-specific r-proteins found nearby
(1405–1,437) h44	S8e; S6e
(176–178,207;209) h9	S8e
(181–205) h9	S4e; S8e
(214–227) h10	S4e
(420–428) h16	none
(440–456) h17	S24e
(561;614–615) h21	none
(841–842)	none
(963–975; 999–1,006) h33	S12e
(1102–1,104;1,109–1,111) h39; (1260) h41	S19e
(1141) h40	S17e
(1504–1,509) 3' extremity	mRNA exit tunnel

The regions indicated in the Table were from Bernier et al. (2018) and Woese (1987). R-proteins are named according to Ban et al. (2014). Numbering refers to the *P. abyssi* 16S rRNA sequence (GenBank AJ248283.1, www.rna.icmb.utexas.edu) also used in PDB 6SWC.

## Archaeal mRNAs

Organization of the archaeal mRNAs is of the bacterial type with many polycistronic genes organized into operons. mRNAs do not have a cap at the 5' end nor a 3' polyadenylated tail. Cryo-EM experiments performed on lysed *Thermococcus kodakaraensis* cells made it possible to observe that most of the polysomes were connected to strands of DNA, thus showing that the mRNA could begin to be translated before its synthesis is complete (French et al., 2007). Hence, from a functional point of view, the prokaryotes archaea and bacteria differ from eukaryotes by the fact that, in the absence of nucleus, transcription and translation take place in the same compartment and that the two processes can, therefore, be coupled (Martin and Koonin, 2006).

Depending on the organism, archaeal mRNAs mainly have Shine-Dalgarno sequences or are mainly leaderless (Dennis, 1997; Ma et al., 2002; Benelli and Londei, 2011). mRNAs are generally considered leaderless if the number of nucleotides preceding the start codon is less or equal to 5 (Babski et al., 2016). Some authors have, however, chosen eight as the threshold, arguing that this is likely the minimal size of an UTR to allow efficient SD-aSD pairing (Jäger et al., 2014). The differences in 5′UTR of mRNAs reflect some diversity in translation initiation mechanisms (Tolstrup et al., 2000; Slupska et al., 2001; Torarinsson et al., 2005; Brenneis et al., 2007; La Teana et al., 2013; Kramer et al., 2014; Schmitt et al., 2019). Recent genome-wide studies, most of them based on differential RNA-seq methods, highlighted mRNA organization in various archaeal branches (Jäger et al., 2009, 2014; Wurtzel et al., 2010; Toffano-Nioche et al., 2013; Li et al., 2015; Babski et al., 2016; Cho et al., 2017; Smollett et al., 2017; Grünberger et al., 2019; Gelsinger et al., 2020). Identification of transcription start points is particularly important in Archaea, where most gene annotations are generated from general computational pipelines that are not fully reliable. Hence, genome-wide transcriptomic studies have made it possible to correct automatic annotation of genomes and some theoretical models directly derived from these annotations.

**Figure 4:**
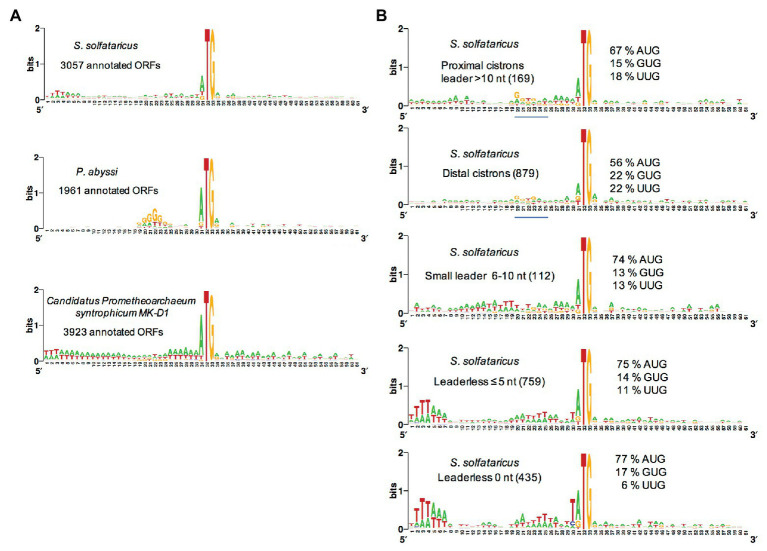
Analysis of translation start regions. **(A)** Analysis of the translation start regions in *Sulfolobus solfataricus*, *P. abyssi*, and the Asgard *Candidatus Prometeoarchaeum syntrophicum*. DNA sequences (60 nt around the first base of the start codon) were extracted from the genomic sequences (She et al., 2001; Cohen et al., 2003; Imachi et al., 2020). Annotations as corrected by Wurtzel et al. (2010) have been used for *S. solfataricus*. Sequence logos were created using Weblogo (Crooks et al., 2004). **(B)** Detailed analysis of the translation start regions in *S. solfataricus*. See also Tolstrup et al. (2000) for an earlier analysis. For each indicated category of transcript (number of ORFs in parentheses), the percentage of AUG, GUG, and UUG start codons are indicated. The position of potential 16S rRNA binding sites (Shine-Dalgarno sequences) in the upper two logos is shown by a blue line. Note that in fully leaderless genes (0 nt), the occurrence of T at the −1 position and the avoidance of UUG as start codon are likely linked to signals for RNA polymerase.

Most euryarchaeal species studied to date mainly harbor Shine-Dalgarno sequences complementary to the 3′ end of their 16S rRNA. Hence, in *Thermococcus onnurineus* (Cho et al., 2017), *Thermococcus kodakarensis* (Jäger et al., 2014), *Methanolobus psychrophilus* (Li et al., 2015), *Methanosarcina mazei* (Jäger et al., 2009), *P. abyssi* (Toffano-Nioche et al., 2013), and *Pyrococcus furiosus* (Grünberger et al., 2019), the abundance of leaderless mRNA is around 15% only. This is in contrast with the high percentage of leaderless mRNA observed in *Saccharolobus solfataricus* (69%; Wurtzel et al., 2010) and *Pyrobaculum aerophilum* (Slupska et al., 2001; Ma et al., 2002), both being crenarchaeaota, and the euryarchaeota *Haloferax volcanii* (72%; Babski et al., 2016; Gelsinger et al., 2020). Interestingly, a quick analysis of the annotated translation initiation regions in the available Lokiarchaeaote genome (Imachi et al., 2020) suggests that SD sequences are not prevalent (Figure 4A).

The availability of a genome-wide transcriptome from *H. volcanii* gave the opportunity to search for features of the many leaderless transcripts (≤5 nt; Babski et al., 2016). First, it was noted that highly transcribed genes typically give leaderless mRNAs. Second, in leaderless mRNAs from abundantly transcribed genes, the AUG start codon was somewhat preferred over GUG. Third, the prevalence of A/G at the first position of the second codon was higher in leadered transcripts than in leaderless ones. However, this analysis did not highlight specific features of leaderless transcripts that may give clues on how they are recognized by the TI machinery. A genome-wide transcriptome is also available for *S. solfataricus* (Wurtzel et al., 2010). Again, comparative sequence logos from transcripts sorted by size of their leaders do not highlight obvious features of leaderless transcripts (Figure 4, see also Tolstrup et al., 2000). In contrast, ORFs that harbor a leader greater than 10 nucleotides, including distal cistrons in operons, show a G/T rich region 10 nucleotides upstream from the start codon, reflecting the occurrence of SD sequences in many cases (Figure 4). This agrees with a recent bioinformatics analysis (Huber et al., 2019), showing that many distal cistrons in overlapping gene pairs carry an SD sequence. Furthermore, the SD motif was found essential for translation of a distal cistron in *S. solfataricus* (Condo et al., 1999). Finally, it is interesting to note that at least in *S. solfataricus*, genes involved in protein translation are over-represented among leadered transcripts (Wurtzel et al., 2010).

The available data suggest that leaderless mRNAs and leadered, SD containing, mRNAs co-exist in almost all archaea, including those for which leaderless mRNAs are prevalent (Tolstrup et al., 2000; Ma et al., 2002; Karlin et al., 2005; Wurtzel et al., 2010; Huber et al., 2019). Moreover, AUG, GUG, and UUG can serve as start codons in all types of mRNAs, whatever the size of the leader. Thus, it is likely that most, if not all, archaea have a TI machinery capable of translating both leaderless and leadered mRNAs (Benelli et al., 2003). This raises the question of the mechanism of mRNAs recruitment by the ribosome.

## SD-Mediated mRNA Recruitment

Sequence analyses show that the 3' extremity of archaeal 16S rRNAs is highly conserved and corresponds to a ^5′^AUCACCUCCU^3′^ consensus (note that crenarchaeota often lack the last CU nucleotides^1^). This sequence is complementary to the SD motif comprising GGAGG. By analogy with bacteria, it can be proposed that in Archaea formation of the SD:antiSD duplex facilitates the recruitment of the small ribosomal subunit. Then, the assembly of the archaeal initiation complex (IC) containing the ternary complex aIF2:GTP:Met-tRNA_i_^Met^ and the two small initiation factors aIF1 and aIF1A is favored. Within this complex, the three initiation factors ensure accurate selection of the start codon (Pedulla et al., 2005; Hasenöhrl et al., 2006, 2009; Gäbel et al., 2013; Coureux et al., 2016, 2020; Monestier et al., 2018).

The role of the SD sequences in translation was experimentally studied in only few archaeal species. Using a cell-free system, the SD motifs were shown essential for translation of a biscistronic mRNA from *S. solfataricus* (Condo et al., 1999). In recent developments of this *S. solfataricus* cell-free system, translation is stimulated by a strong SD motif placed ahead of the start codon of the chosen gene (Lo Gullo et al., 2019). The GGAGGUCA SD motif of the *gvp*H gene from *Halobacterium salinarium*, involved in the gas vesicle formation, was shown to enhance translation efficiency using an *in vivo* assay in the related halophilic archaeon *H. volcanii* (Sartorius-Neef and Pfeifer, 2004). However, in *H. volcanii* (72% leaderless mRNAs), the SD motif was shown to be non-functional in translation initiation of the monocistronic *sod* mRNA (Kramer et al., 2014). Moreover, in this halophilic archaea, translational coupling was demonstrated for overlapping gene pairs. In this case, the SD motif in the distal cistrons appeared more important for reinitiation than for *de novo* initiation (Huber et al., 2019). This raises the possibility that distal cistrons in overlapping gene pairs are translated by a mechanism where 70S ribosomes terminate and then reinitiate without dissociation. Alternatively, the terminating ribosome may dissociate but the SSU would remain bound to the mRNA thanks to the SD sequence (Huber et al., 2019). It should, however, be noted that translation of an SD-leadered distal cistron in a *S. solfataricus* cell-free system was found to be independent of the translation of the first cistron (Condo et al., 1999). Notably, *S. solfataricus* and *H. volcanii* both are organisms that widely use leaderless mRNAs. It cannot be excluded that in these organisms, TI mechanism evolved in such a way that interaction of the SD motif with the antiSD sequence of the 16S rRNA became less important for the stability of the TI complex. Unfortunately, to our knowledge, the role of the SD sequence in TI efficiency was not studied *in vivo* in euryarchaea, where a SD sequence is found in the major parts of the genes. Nevertheless, recent structural studies showing the formation of an SD:antiSD duplex in the mRNA exit chamber of the SSU of *P. abyssi* (71% SD-leadered genes; Ma et al., 2002; Toffano-Nioche et al., 2013) strongly suggest that as in bacteria, the SD motif stabilizes the TI complex.

## The SD Duplex is Bound in an mRNA Exit Channel That Differs From That of Bacteria

**Figure 5:**
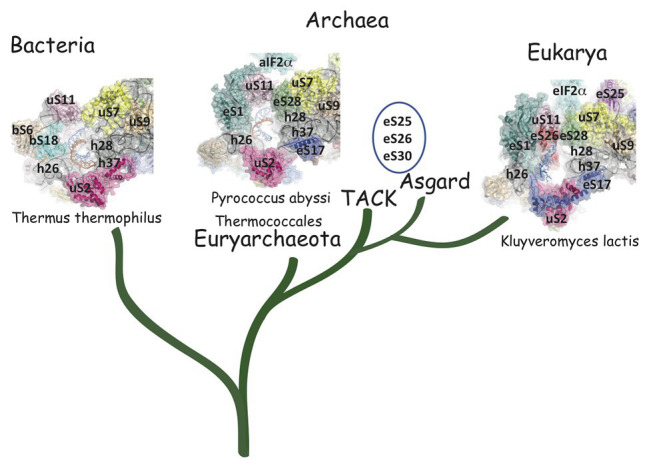
Comparison of the mRNA exit channels in the three domains of life. Surface representations of the mRNA exit channels of representative structures in the three domains of life. The mRNA is shown in blue and the 3'extremity of the rRNA is shown in orange. R-proteins are labeled using the Ban et al. (2014) nomenclature. The figure illustrates the similarity of the archaeal and eukaryotic mRNA exit channels vs. the bacterial channel. TACK and Asgard Archaea have three additional proteins in their SSU as compared to thermococcales (eS25, eS26, and eS30). The structures are from PDB 4VY4 (Yusupova et al., 2006), PDB 6SWC (Coureux et al., 2020), and PDB 6FYX (Llacer et al., 2018). The figure is adapted from Coureux et al. (2020).

The cryo-EM structure of a TI complex from *P. abyssi* (Pa) using an mRNA derived from that of the gene coding for Pa-aEF1A containing a strong SD sequence [A(−17)UUUGGAGGUGAUUUAAA(+1)UGCCAAAG(+9)] is known at 3.4 Å resolution (Coureux et al., 2020). In the mRNA exit chamber, the SD duplex is extended to nine nucleotides and involves the ^5′^AUCACCUCC^3′^ sequence of the 3′-end of the 16S rRNA. The SD helix is positioned in the mRNA exit chamber delineated by uS11, eS1, and h26 on the one side and by uS7, eS28, h28, and h37, on the other side (Figure 5). Interactions of uS11 with eS28 and uS7 connect the platform to the head and form the SD duplex channel. uS2 and eS17 are located at the end of the mRNA exit chamber. The archaeal mRNA exit chamber was compared to the bacterial one. As shown in Figure 5, bS6 and bS18 are found in place of eS1 in the bacterial ribosome. In bacteria, eS28 is absent and uS2 possesses a supplementary inserted helical domain occupying the position of eS17. Interestingly, comparison of the bacterial structures with the archaeal one showed that the spacing between the AUG codon and the SD sequence changed the position of the duplex in the chamber, probably explaining how it influences translation initiation efficiency (Coureux et al., 2020). Overall, archaeal and bacterial exit channels appear as two structural solutions for binding the SD duplex. These two solutions reflect an early divergence of the ribosomes from these two domains (Figure 5).

The mRNA exit tunnel of the euryarchaeota *P. abyssi* is of the eukaryotic type (Figure 5). Notable differences are, however, observed. First, in the yeast ribosome, eS17 has a long C-terminal extension contacting the mRNA and second, eS26 stabilizes the 5′ end of the mRNA (Llacer et al., 2018; Simonetti et al., 2020). Interestingly, eS26 was proposed to be involved in recognition of Kozak sequence elements (Ferretti et al., 2017). Importantly, in eukaryotes, initiation factors were shown to be involved in stabilization of the mRNA in the exit channel. Indeed, Kozak consensus nucleotides are recognized in the E site by domain 1 of the α subunit of eIF2. Such an interaction was not observed with the homologous protein aIF2α in the *P. abyssi* complex (Coureux et al., 2020). In addition, the eukaryotic-specific eIF3a subunit would also stabilize the mRNA at the exit channel pore (Llacer et al., 2018). These differences illustrate how eukaryotic and thermococcal ribosomes evolved the mRNA binding modes in the exit pocket, in relation with the canonical eukaryotic scanning mode vs. the SD-assisted AUG recognition mode occurring in many genes in the archaeal domain. In this view, it is notable that eS26 is absent in euryarchaeotes but present in TACK/Asgard genomes (Lecompte et al., 2002; Schutz et al., 2018). Because the archaeal version of the exit chamber is a simplified version of the eukaryotic one, this argues in favor of the controversial hypothesis that eukaryotic ribosomes have evolved from within the archaeal version (Zaremba-Niedzwiedzka et al., 2017; Eme and Ettema, 2018).

## Start Codon Selection Mechanism

Three archaeal initiation factors, aIF1, aIF1A, and aIF2, participate in start codon selection on the SSU. The biochemical and structural data concerning these factors have been recently reviewed (Schmitt et al., 2019). Briefly, aIF1 is a small protein of ca. 100 residues that binds to the 30S in front of the P site (Coureux et al., 2016). Biochemical data using *P. abyssi* and *S. solfataricus* aIF1 have shown that the factor favored mRNA binding and formation of the initiation complex (Hasenöhrl et al., 2006, 2009; Monestier et al., 2018). The factor was also shown to induce a dynamic character of the IC favoring proofreading of erroneous initiation complexes (Hasenöhrl et al., 2009; Monestier et al., 2018). The role of aIF1 in translation fidelity is consistent with that observed for eIF1 in eukaryotes (Algire et al., 2005; Nanda et al., 2009). This function is also reminiscent of that of the bacterial translation initiation factor IF3 whose C-terminal domain has some structural resemblance with e/aIF1 (Figure 2; Rodnina, 2018). Notably, IF3 C-terminal domain alone is sufficient to sustain the growth of an IF3-deficient *E. coli* strain (Ayyub et al., 2017).

**Figure 6:**
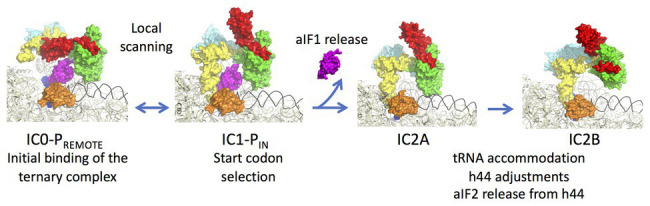
Steps of translation initiation in *P. abyssi*. Surface representation of successive *P. abyssi* translation initiation complexes. aIF2, aIF1, and aIF1A are shown with the same color code as in Figure 2. mRNA is in dark blue, initiator tRNA is in yellow, and the h44 helix is in black. The figure shows start codon selection in the full archaeal TI complex where IC0-P_REMOTE_ and IC1-P_IN_ are in equilibrium until a start codon is found in the P site (Coureux et al., 2016). Codon:anticodon pairing stabilizes the IC1 state and triggers aIF1 release. In IC2A, the initiator tRNA fully accommodates. Release of aIF1 would cause both Pi release from aIF2γ and h44 adjustments leading to irreversible aIF2 release. The figure is adapted from Coureux et al. (2020).

aIF1A is a small protein of ca. 100 residues that contains an OB-fold. Like its eukaryotic homologue, the factor occupies the A site on the SSU. Importantly, the eukaryotic version of the factor contains N and C-terminal extensions necessary for the scanning of the PIC along the mRNA (Figure 2; Pestova et al., 1998). aIF2 is a heterotrimeric protein that binds Met-tRNA_i_^Met^ in a GTP dependent manner (Yatime et al., 2004, 2006; Pedulla et al., 2005; Sokabe et al., 2006; Stolboushkina et al., 2008). γ is the core subunit that binds GTP (Schmitt et al., 2002; Dubiez et al., 2015). α and β are bound to γ but do not interact together (Yatime et al., 2004; Schmitt et al., 2010). A 5 Å crystal structure of the TC (aIF2:GDPNP:Met-tRNA) showed that the initiator tRNA is bound to aIF2 *via* the C-terminal domain of α and the domains I and II of γ, while the aIF2β subunit did not strongly contribute to the tRNA binding (Schmitt et al., 2012). A cryo-EM study of an archaeal initiation complex from *P. abyssi* containing the three initiation factors showed two conformations (Coureux et al., 2016). Analysis of these two conformations led to a model of start codon selection. In the major conformation, called IC0-P_REMOTE_, the anticodon stem-loop of the Met-tRNA_i_^Met^ is out of the P site. aIF2γ is bound to helix h44 and interacts with aIF1. The N-terminal domain of aIF1 would contact the two switch regions that control the nucleotide state of aIF2γ. In the second conformation, called IC1-P_IN_, the anticodon stem-loop of the Met-tRNA_i_^Met^ is bound to the P site, while the position of aIF2γ on h44 has not changed. aIF1A is still bound within the A site and aIF1 still located in front of the P site. The IC0-P_REMOTE_ and IC1-P_IN_ positions are in equilibrium and the transition from one position to the other, accompanied by a 30S head motion, reflects the dynamics of the PIC during start codon selection in the P site (Coureux et al., 2016; Monestier et al., 2018). As observed for eukaryotic PIC (Lomakin et al., 2003; Weisser et al., 2013; Llacer et al., 2015), definitive stabilization of the tRNA in the P site is impaired by aIF1. This is consistent with the function of aIF1 in destabilization of erroneous TI complexes (Hasenöhrl et al., 2009; Hussain et al., 2014; Llacer et al., 2015; Monestier et al., 2018). Moreover, interaction of aIF2 with h44 of the 30S also counteracts final accommodation of the tRNA in the P site unless the start codon is base-paired with the tRNA anticodon. Indeed, codon-anticodon pairing compensates for the restoring force exerted on the tRNA by aIF2 because of its interaction with h44. Such a compensation would allow a longer stay of the initiator tRNA in the P site and trigger further events, including aIF1 departure because of steric hindrance with the tRNA, and release of aIF2 in its GDP bound form (Figure 6).

In order to better understand the role of aIF1 in the mechanism, an IC2 complex made in its absence was studied by cryo-EM at 3.4 Å resolution. Consistent with the above ideas, the IC2 complex shows stable accommodation of the initiator tRNA in the P site (Monestier et al., 2018; Coureux et al., 2020). Comparison of all states (Figure 6) suggests that a first set of conformational adjustments of h44 accompanies aIF1 departure causing in turn the release of its contacts with aIF2. These events would lead to the release of aIF2-GDP. Re-adjustments of the position of h44 in the bulge region could explain how the contacts between h44 and aIF2γ are lost. Both these h44 movements and the release of contacts between aIF1 and aIF2γ could explain how aIF2 is detached from the ribosome after start codon recognition and aIF1 release. In eukaryotes, it was shown that full release of eIF2 is linked to the release of Pi coming from GTP hydrolysis on eIF2 (Algire et al., 2005). In archaea, the breakdown of contacts between the N-terminal domain of aIF1 and the switch regions of aIF2γ could explain the coupling between aIF1 release and Pi release.

Overall, the available data suggest similarity in the mechanisms involved in start codon selection by e/aIF1, e/aIF1A, and e/aIF2 on the SSU in eukaryotes and archaea.

## Three Universal Proteins Participate in the Selection of the Initiator tRNA at the P Site

**Figure 7:**
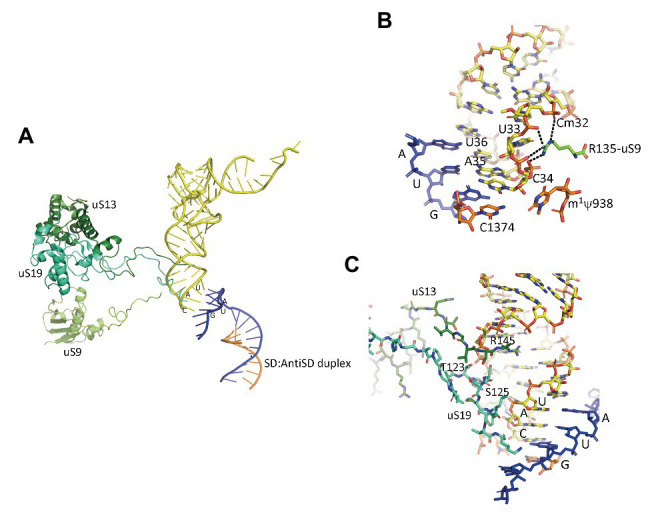
Interaction of the initiator tRNA at the P site with the universal proteins uS9, uS13, and uS19. **(A)** Overall view of the accommodated tRNA as observed in the IC2 complex from *P. abyssi* (Coureux et al., 2016). The color code is the same as in Figure 6. **(B)** Close up showing the interaction of the strictly conserved terminal arginine of uS9 with the codon:anticodon duplex. **(C)** Close up showing the interaction of the uS13 and uS19 tails with the major groove of the anticodon stem-loop of the initiator tRNA.

In the IC2 complex, the anticodon stem of the tRNA is tightly bound to the P site. A network of interactions involving the C-terminal tails of the three universal proteins uS9, uS13, and uS19 is observed (Figure 7). The C-terminal arginine R135 of uS9 is hydrogen bonded to the phosphate groups of Cm32, U33, A35, of the initiator tRNA. This interaction is also observed in eukaryotes (Llacer et al., 2018) and bacteria (Figure 7; Selmer et al., 2006; Fischer et al., 2015; Polikanov et al., 2015; Hussain et al., 2016). In the *P. abyssi* IC, the position of the C-terminal arginine uS9-R135 is further stabilized by interaction with uS19-R124. uS9 is highly conserved in the three domains of life and the protein systematically ends with an arginine residue (see alignments in Melnikov et al., 2018). The role of uS9 C-tail in fidelity was previously shown by studies with bacterial (Hoang et al., 2004; Noller et al., 2005; Arora et al., 2013a,b) and yeast systems (Ghosh et al., 2014; Jindal et al., 2019). Moreover, in eukaryotes, uS9 favors the recruitment of the TC on the ribosome (Jindal et al., 2019). Thus, the IC2 structure indicates a universal involvement of uS9 tail in the fidelity of TI. The constant role of the C-terminal arginine of uS9 would, therefore, have been acquired very early in evolution.

**Figure 8:**
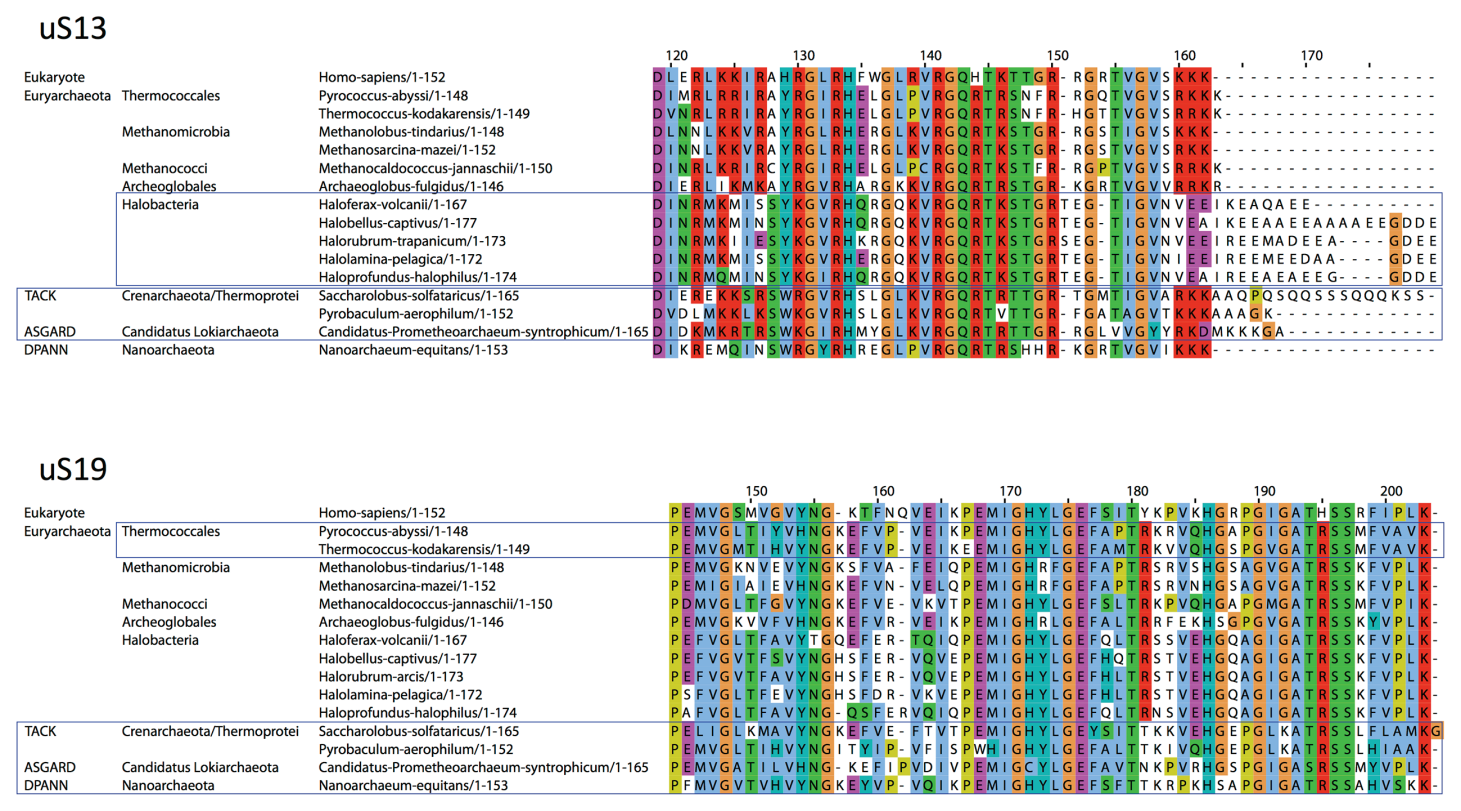
Sequence alignments of the uS13 and uS19 C-terminal tails. uS13 and uS19 sequences were extracted and aligned using Pipealign (Plewniak et al., 2003). After visual inspection, several families regarding the C-terminal tail specificities in Archaea were identified. For uS13, ca. 250 archaeal sequences were used to which we added manually 100 sequences from halobacteria. For uS19, ca. 600 archaeal sequences were used. Typical representatives of each family are shown. The *Homo sapiens* sequence is used as a eukaryotic reference for comparison.

Concerning uS13 and uS19, the C-tails of these two proteins are oriented toward the major groove of the anticodon stem of the initiator tRNA (Figure 7). In particular, they interact with G30 of the second base pair of the almost universally conserved three GC base pairs of the anticodon stem of the initiator tRNA, that play a crucial role in translation fidelity (Samhita et al., 2012; Hussain et al., 2016; Shetty et al., 2017; Ayyub et al., 2018). Sequence alignments of uS13 showed that the basic character of the C-tail is conserved in the three domains of life. Sequence conservation of the C-tail, though strong, is less strict than that of uS9, with some organisms, in all domains, having variable tail lengths (Melnikov et al., 2018). We refined this analysis by focusing on the uS13 tails in archaeal branches. It is striking that the tails have evolved in a branch-dependent manner (Figure 8). Halobacteria strongly differentiate by an acidic tail. On another hand, TACK and Asgard frequently display tails of variable lengths reminiscent of low complexity regions identified in various prokaryotic proteins (Ntountoumi et al., 2019). The C-tail of uS19 is very basic in bacteria. In archaea and in eukaryotes, the tails are eight residues longer with a less pronounced basic character (see alignments in Melnikov et al., 2018). Again, sequence alignments of archaeal representatives (Figure 8) highlight some branch specificities. Overall, the variations of the tails of uS13 and uS19 in archaea likely reflect tuning of translation mechanisms to peculiar environmental conditions.

Consistent with the archaeal case, the tails of uS13 and uS19 were recently observed in contact with the anticodon stem of the initiator tRNA in a mammalian late-stage initiation complex (Simonetti et al., 2020). On another hand, uS13 and uS19 are also involved in translation elongation, as observed recently in a mammalian elongation complex (Bhaskar et al., 2020) and during the translocation step in bacteria (Zhou et al., 2013).

**Figure 9:**
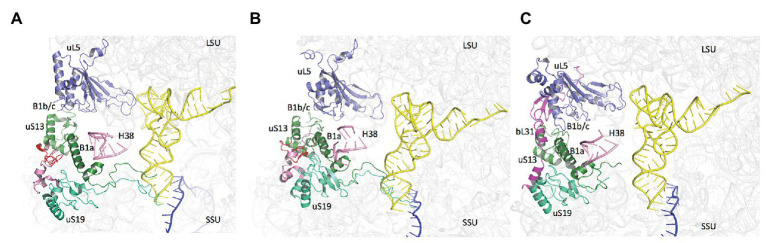
Domain specificities of uS13 and uS19 cores. The three panels show the B1a-B1b/c bridge. **(A)** Archaeal case. The view is a composite using the SSU from PDB 6SWC (Coureux et al., 2020) and the LSU from PDB 4V6U (Armache et al., 2013). **(B)** Structure of human ribosome from PDB 6Y0G (Bhaskar et al., 2020). **(C)** Structure of *Escherichia coli* ribosome from PDB 5AFI (Fischer et al., 2015). The views show that archaeal and eukaryotic uS13 and uS19 have specific extensions (in red and pink, respectively) that contribute to the intersubunit bridge. Bacteria have instead a specific ribosomal protein bL31.

Notably, the core domains of uS13 and uS19, located on the SSU head, are involved in the B1a and B1b/c bridges with the large ribosomal subunit (LSU). Several studies in yeast identified allosteric information pathways connecting functional centers in the LSU to the decoding center in the SSU through these bridges (Ben-Shem et al., 2011; Rhodin and Dinman, 2011; Bowen et al., 2015; Bhaskar et al., 2020). Interestingly, archaeal and eukaryotic uS13 and uS19 have sequence insertions in their core domains as compared to the bacterial proteins. These insertions expand the contact area between the two proteins (Figure 9). In bacteria, where the two insertions are missing, only few contacts between uS13 and uS19 are visible. However, a bacterial specific protein, bL31, interacting with uS19 was recently shown bridging the two subunits of the ribosome (Figure 9; Fischer et al., 2015). This bL31 protein could ensure a similar function as the two specific eukaryotic and archaeal extensions of uS13 and uS19. Overall, these observations likely reflect late evolutions of the mechanisms in the three domains of life. The eukaryotic and archaeal C-tails and insertions further argue in favor of the archaeal ribosome being of the eukaryotic-type.

## Involvement of Base Modifications in Start Codon Selection

Like in bacteria and eukaryotes, a series of rRNA modifications is observed around the P site (Coureux et al., 2020). Some of these rRNA modifications are classified as universally conserved. They correspond to m^3^U1467 (m^3^U1498, *E. coli* numbering) and the two dimethyladenosines m_2_^6^A1487, m_2_^6^A1488 (m_2_^6^A1518, m_2_^6^A1519 in *E. coli*). In contrast, for other positions, the pattern of rRNA modification is specific to eukaryotes and archaea (Coureux et al., 2020). A first layer of rRNA modification stabilizes the codon:anticodon duplex. A second layer made up m^6^_2_A1487, m^6^_2_1488, hm^5^C1378, stabilizes the first layer. Notably, the N-terminal part of eL41 contacts several modified bases linked to the P site. In particular, the conserved R15 (*P. abyssi* numbering) interacts with an acetylcytidine residue (Ac^4^C1476, *P. abyssi* numbering). The case of eL41 is rather intriguing. Indeed, this protein was first identified as a protein belonging to the large ribosomal subunit explaining its naming. However, recent structures of eukaryotic and archaeal ribosomes revealed that the protein mainly interacts with the SSU. Because the protein interacts with the network of modified bases involved in the control of start codon selection, eL41 was proposed to be involved in the regulation of the process. Up to now, eL41 has been found in eukaryotes and in most archaea (Lecompte et al., 2002; Coureux et al., 2020). Nevertheless, its identification is rendered difficult because of the small size of the protein (25–37 residues). Finally, it is notable that *P. abyssi* and *T. kodakarensis* ribosomes contain a large amount of ac^4^C, likely involved in thermostability (Coureux et al., 2020; Sas-Chen et al., 2020). Indeed, the amount of ac^4^C varies with growth temperature (Sas-Chen et al., 2020).

## Late Steps of Translation Initiation

In eukaryotes and in archaea, the late stage of TI occurring after e/aIF2 departure involves the two factors e/aIF1A and e/aIF5B. These two factors ensure final check of the presence of the initiator tRNA and assembly with the LSU (Figure 1; Choi et al., 1998; Pestova et al., 2000; Maone et al., 2007). As other translational GTP-ases, e/aIF5B activity is related to the transition between an active GTP state and an inactive GDP state controlled by the movement of two switch regions (switch 1 and switch 2) that interact with the nucleotide. aIF5B is composed of four domains (Figure 2). The structure of aIF5B from *Methanobacterium thermoautotrophicum* showed that domains I (GTP binding domain), II, and III are packed together and linked by a long α-helix (helix h12) to domain IV (Figure 2; Roll-Mecak et al., 2004). Domain IV contains a β-barrel and is responsible for the binding of the methionylated-CCA end of the initiator tRNA (Guillon et al., 2005). In eukaryotes, the integrity of the h12 helix and the multidomain nature of the factor were shown important for its function (Shin et al., 2011; Kuhle and Ficner, 2014; Huang and Fernández, 2020). Eukaryotic eIF5B contains an additional N-domain which displays little sequence conservation and was shown to be dispensable in yeast (Shin et al., 2002). eIF5B was shown to directly interact with the eukaryotic-specific C-tail of eIF1A (Choi et al., 2000; Marintchev et al., 2003; Zheng et al., 2014). This interaction is required for efficient subunit joining (Acker et al., 2006). Such an interaction between aIF1A and aIF5B has not been evidenced in archaea. Possibly, the two proteins do not directly interact because aIF1A does not possess the C-terminal extension and because aIF5B presents a supplementary helix at the position expected for aIF1A binding site (Murakami et al., 2016).

e/aIF5B-GTP binds the SSU (Maone et al., 2007) and accelerates the recruitment of the large ribosomal subunit (Pestova et al., 2000; Acker et al., 2009). It is possible that interaction of e/aIF5B with proteins of the P stalk contribute to favor the binding of the SSU-IC to the LSU (Murakami et al., 2018). The position of eIF5B on the 80S has been observed in several Cryo-EM studies (Fernandez et al., 2013; Yamamoto et al., 2014; Huang and Fernández, 2020) with domain IV holding the Met-CCA extremity of the initiator tRNA. In addition, the dynamics of its binding has been studied in real-time single-molecule experiments (Wang et al., 2019). A rearrangement of the 80-IC complex containing eIF5B would trigger GTP hydrolysis and the release of the factor leading to the formation of a ribosome competent for elongation.

Importantly, e/aIF5B and e/aIF1A are orthologues of the bacterial proteins IF2 and IF1, respectively. The assembly step has, therefore, a universal character. In bacteria, evolution might have selected formylation of the initiator tRNA to enhance specificity, whereas this improvement would have been gained in eukaryotes and archaea thanks to the emergence of e/aIF2. Interestingly, several studies have shown that in some non-canonical cases, eukaryotic translation initiation used eIF5B instead of eIF2 for the recruitment of the initiator tRNA (Terenin et al., 2008; Thakor and Holcik, 2012; Ho et al., 2018; Ross et al., 2018). This argues in favor of an ancestral translation initiation mechanism involving e/aIF5B/IF2 and e/aIF1A/IF1 that could have been used in the last common universal ancestor (LUCA) and that should also be discussed in the light of what is known for translation initiation of leaderless mRNAs (Londei, 2005; Beck and Moll, 2018).

## Translation Initiation of Leaderless mRNAs

### Insights From Bacteria

The mechanisms for initiation of learderless mRNAs translation in archaea have been addressed in a limited number of reports. Because leaderless mRNAs are found in the three domains of life (Janssen, 1993), some data concerning bacteria, and to a lesser extent mitochondria may be relevant to the archaeal case. Leaderless mRNAs can indeed be translated in *E. coli* (Balakin et al., 1992; Wu and Janssen, 1996; Grill et al., 2000; Moll et al., 2002b; Udagawa et al., 2004; Vesper et al., 2011; Yamamoto et al., 2016; Beck and Moll, 2018) and are even widespread in some bacterial species such as *Deinococcus* species (de Groot et al., 2014; Bouthier de la Tour et al., 2015) and mycobacterial species (Cortes et al., 2013; Shell et al., 2015; Li et al., 2017).

In bacteria and in Archaea, leaderless mRNAs are featured by a 5′ tri-phosphate and an AUG (or GUG or UUG) start codon near the 5′ extremity. Notably, in mammalian mitochondria, post-transcriptional processing of long transcript produces a free phosphate group at the 5′ end of the mRNA. Accordingly, whereas in bacteria, a free phosphate group at the 5′ end of a leaderless mRNA was shown important for TI (Giliberti et al., 2012), this is not the case in mitochondria (Christian and Spremulli, 2010). Finally, in *E. coli*, sequences located dowstream to the start codon, called “downstream box” were proposed to contribute to TI efficiency of leaderless mRNAs (Sprengart et al., 1996; Martin-Farmer and Janssen, 1999) although this point is debated (Resch et al., 1996; O'Connor et al., 1999).

Studies in *E. coli* mainly used the mRNA encoding the cI repressor of the λ bacteriophage as a model leaderless mRNA and its translation from assembled 70S ribosomes was early proposed (Balakin et al., 1992). Numerous studies have demonstrated that 70S monomers were able to initiate translation of leaderless mRNAs *in vitro* (e.g., Moll et al., 2004; Udagawa et al., 2004; Yamamoto et al., 2016). Moreover, it was observed that in a strain deficient for the ribosome recycling factor RRF, i.e., under conditions where 70S ribosomes were abundant, translation of leaderless mRNAs was maintained whereas that of SD-leadered mRNAs was inhibited (Moll et al., 2004). The possibility for the 70S monomers to initiate translation of leaderless mRNAs is connected to the accessibility of the ribosome for the mRNA (Yamamoto et al., 2016). Indeed, the channel in the 30S subunit can readily bind any mRNA at an internal site whereas a 70S ribosome must thread the mRNA from an extremity. Notably, initiation with 30S subunits has also been observed (Balakin et al., 1992; Grill et al., 2000).

In bacteria, the initiator tRNA and initiation factor 2 (IF2) have a crucial role in the recruitment of the leaderless mRNA, whether on the 30S subunit (Grill et al., 2000, 2001) or on the 70S ribosome (Udagawa et al., 2004; Yamamoto et al., 2016). This strongly indicates that codon:anticodon base pairing has an important contribution to the affinity of the leaderless mRNA for the ribosome. Notably, the 5′-triphosphate group in the vicinity of the start codon may also be useful for leaderless mRNA binding (Giliberti et al., 2012). In this context, it is notable that the addition of 5 or 10 nucleotides without an SD sequence before the AUG codon abolished 70S initiation *in vitro* (Udagawa et al., 2004). IF3 was also shown important for TI of leaderless mRNAs though its role is less clear. In a purified translation system, leaderless mRNA translation was found strictly dependent on IF3 (Yamamoto et al., 2016). However, IF3 was also reported to be inhibitory, in particular at high concentrations (Tedin et al., 1999; Grill et al., 2001; Udagawa et al., 2004). The inhibitory effect of IF3 may be linked to its ribosomal subunits anti-associative activity (Dallas and Noller, 2001) and to its possible role in ribosome recycling (Peske et al., 2005; Zavialov et al., 2005). Hence, prolonged incubation with high levels of IF3 may decrease the availability of 70S ribosomes (Peske et al., 2005). Further, IF3 closely participates in start codon selection by destabilizing codon-anticodon interaction (Hartz et al., 1989; Gualerzi and Pon, 1990). Thus, because of the key importance of codon-anticodon pairing in the binding of a leaderless mRNA by the initiating ribosome, translation of leaderless mRNAs may be disfavored by high IF3 levels.

In summary, the most recent data rather favor translation initiation of leaderless mRNA in bacteria with 70S ribosomes, assisted by IF2, f-Met-tRNA_f_^Met^, and IF3. However, the co-existence of a mechanism using 30S subunits cannot be fully excluded at this stage.

Specialized ribosomes were observed to translate leadered and leaderless mRNAs with different efficiencies. Notably, the absence of bS1 and uS2 favors leaderless mRNA translation by 70S ribosome (Moll et al., 2002a). Further, exposure of *E. coli* to the antibiotic kasugamycin induced 61S particles, devoid of six proteins in the small subunit (bS1, uS2, bS6, uS12, bS18, and bS21), that selectively translate leaderless mRNAs (Kaberdina et al., 2009). Finally, MazEF, a toxin-antitoxin system induced by stress in *E. coli* was shown to function by generating specific leaderless mRNAs together with specialized ribosomes lacking the 16S rRNA region containing the anti-SD motif (Vesper et al., 2011).

Another example of leaderless translation is found in mammalian mitochondria. In these organelles, IF1 is absent whereas mt-IF2 and mt-IF3 have acquired specific structural extensions. Notably, mammalian mt-IF2 can replace both IF1 and IF2 for supporting *E. coli* growth (Gaur et al., 2008). Recent structural studies proposed that mt-IF3 would be necessary to stabilize the mt-SSU head for the accommodation of mt-IF2. After mt-IF3 release the recruitments of the initiator tRNA, of the mRNA and of the LSU would complete the initiation complex. Release of mt-IF3 would be an obligatory step for tRNA binding and mt-IF2 would be necessary for leaderless mRNA recruitment by the ribosome (Kummer et al., 2018; Koripella et al., 2019; Khawaja et al., 2020).

### The Archaeal Case

In archaea, recruitment of leaderless mRNAs by the ribosome has been studied *in vitro* using *S. solfataricus* components (Condo et al., 1999; Benelli et al., 2003). It was observed that 30S subunits were unable to stably bind leaderless mRNA. However, addition of methionylated initiator tRNA was sufficient to form a complex, where the 30S subunit is positioned with the start codon of the leaderless mRNA in the P site, as assessed by toeprinting methods (Benelli et al., 2003). Further addition of aIF5B, the homologue of bacterial IF2 (Figure 2) did not significantly enhance the intensity of the toeprint (Benelli et al., 2003). Whether initiation of archaeal leaderless mRNAs translation occurs with 30S subunits or 70S ribosomes remains an open question. Considering the properties of archaeal initiation factors, several possibilities may be envisaged. In the first model, initiation would occur with 70S subunits. In this case, a direct involvement of the heterotrimeric aIF2 in tRNA recruitment is unlikely because the structure of the complex containing the 30S subunit, aIF2, and the initiator tRNA is not compatible with 50S assembly (Armache et al., 2013; Coureux et al., 2016, 2020). However, aIF5B, the archaeal homologue of bacterial IF2, can indeed bind both the 70S ribosome (Maone et al., 2007) and the methionylated initiator tRNA (Guillon et al., 2005). Thus, a 70S:aIF5B:Met-tRNA_i_^Met^ complex would be able to recruit the leaderless mRNA thanks to start codon-tRNA anticodon pairing and possibly binding of the 5′-triphosphate on the mRNA. A second possible mechanism would be mediated by the SSU, similarly to the SD-leadered mRNAs (see above). In such a model, the mRNA would be mainly tethered to the 30S subunit thanks to base pairing of the start codon with the anticodon of the initiator tRNA bound to aIF2-GTP and to the P site (Benelli et al., 2003). Whatever the mechanism, recruitment of the leaderless mRNA by either 30S or 70S ribosomes, it may be imagined that the leaderless mRNA can interact with the ribosome apart from the codon-anticodon pairing. In this view, it should be reminded that the γ subunit of *S. solfataricus* aIF2 is able to bind the 5′-triphosphorylated end of mRNAs, thereby protecting them against degradation (Hasenöhrl et al., 2008). It cannot be excluded that this activity also facilitates leaderless mRNA recruitment by the ribosome. Furthermore, as discussed in the present review (see Figure 5), the mRNA exit channel on the 30S subunit has archaeal-specific features, as well as features distinguishing archaeal branches. For instance, *S. solfataricus* and *P. aerophilum*, two archaea widely using leaderless mRNAs, have a larger set of 30S r-proteins. In contrast, Halobacteria and Thermoplasma, also widely use leaderless mRNAs but have a reduced set of r-proteins in the 30S (Lecompte et al., 2002). This opens the possibility that mechanisms of leaderless mRNA recruitment may somewhat vary within the archaeal world. These considerations deserve further investigation.

Finally, it has been reported that mRNAs with leaders not including an SD sequence were not translated in a cell-free *S. solfataricus* system, where leaderless mRNAs were efficiently used (Benelli et al., 2003). Leadered mRNAs with no obvious SD sequence apparently occur (Wurtzel et al., 2010). It may be hypothesized that translation of such mRNAs requires scanning of a 70S ribosome from the 5′-end of the mRNA, as proposed in the bacterial case (Yamamoto et al., 2016). However, the mechanistic analogy between the bacterial and archaeal cases is limited by the observation that 70S-bound IF3 is mandatory for scanning in bacteria (Yamamoto et al., 2016). Whether aIF1 might play a similar role remains an open question. Hence, another possibility would be a mechanism involving an IC on the archaeal 30S resembling and foreshadowing eukaryotic scanning. Further studies are clearly needed to assess these various hypotheses or to decipher alternative mechanisms.

## Concluding Remarks

Study of TI mechanisms in Archaea has gained a new momentum in recent years thanks to the fast development of phylogenetic analyses, genome-wide studies, and 3D structure determinations. The archaeal ribosomes are of the eukaryotic type but they have specificities linked to the mode of mRNA recruitment. In many archaea, mRNAs carry SD sequences complementary to the 3′ end of the ribosomal RNA. The formation of the SD:antiSD duplex favors the initiation complex and positioning of the initiator tRNA in the vicinity of the P site on the SSU. The SD:antiSD duplex is stabilized in an exit chamber on the SSU. The protein organization of this chamber is specific to the archaeal domain. It is very close to that in eukaryotes but very different from that in bacteria. Thus, bacteria and archaea have evolved two different structural solutions for the binding of the SD:antiSD duplex.

On another hand, transcriptomic data show that in many archaea, mRNAs are leardeless. Thus, the mode of recruitment of these leaderless mRNAs would be different. Leaderless mRNAs are considered to be ancient and may reflect the original TI mechanisms that existed in the LUCA. However, translation initiation mechanisms of these mRNAs are still unclear and the current data do not exclude an initiation mode involving the 30S or a pre-assembled 70S. In both cases, the role of the initiator tRNA would be essential. Interestingly, the presence of the three ribosomal proteins, eS26, eS25, and eS30, systematically found in eukaryotes, varies between the different branches of archaea. These three proteins have a direct link with translation initiation. In eukaryotes, eS26 is located in the mRNA exit channel, eS30 is located in the mRNA entry channel, and eS25 is observed in contact with the initiator tRNA. Additional studies are necessary to establish their function in Archaea. However, it is tempting to imagine that the presence of these proteins is related to an evolution of the ribosomes coupled to that of the organization of mRNAs. To answer these questions, the accumulation of new data is necessary. In this context, high-resolution cryo-EM structures will contribute to bring important information.

## Author Contributions

All authors listed have made a substantial, direct and intellectual contribution to the work, and approved it for publication.

### Conflict of Interest

The authors declare that the research was conducted in the absence of any commercial or financial relationships that could be construed as a potential conflict of interest.

## Footnotes

^1^http://www.rna.icmb.utexas.edu/

## Abbreviations

TI: Translation initiation
SSU: Small ribosomal subunit
LSU: Large ribosomal subunit
IC: Initiation complex
TC: Ternary complex e/aIF2:GTP:Met-tRNA_i_^Met^
LUCA: Last universal common ancestor.
